# Moving along the ALS‐bvFTD spectrum: changes in MEG‐based brain network topology

**DOI:** 10.1002/alz.087328

**Published:** 2025-01-03

**Authors:** Rosanne Govaarts

**Affiliations:** ^1^ Amsterdam UMC, Amsterdam, Amsterdam Netherlands

## Abstract

**Background:**

Amyotrophic lateral sclerosis (ALS) with only motor impairment (ALS‐pure motor) and the behavioral variant of frontotemporal dementia (bvFTD) are hypothesized to be the extreme ends of the ALS‐bvFTD spectrum. This spectrum also encompasses ALS patients with mild to severe cognitive impairment (ALSci) and/or behavioral impairment (ALSbi), including ALS with concomitant bvFTD.

In a previous study, using magnetoencephalography (MEG), in early symptomatic ALS patients we showed resting‐state functional connectivity changes in frontal, limbic and subcortical regions that overlap considerably with bvFTD. These findings showed the potential of MEG to detect brain changes in early symptomatic phases of ALS and contribute to our understanding of the disease spectrum, with ALS and bvFTD at the two extreme ends.

In our current study we used MEG in a longitudinal design, to investigate the changes in brain network topology of ALSci/bi over time as compared to ALS‐pure motor and bvFTD patients.

**Method:**

Resting‐state MEG was recorded in ALS‐pure motor (n = 9), ALSci/bi (n = 16) and bvFTD (n = 16) at baseline and mean (SD) 5.4‐month (0.8) follow‐up, and projected to source‐space. The corrected version of the amplitude envelope correlation (AECc) was applied to compute frequency‐band specific functional connectivity between brain regions, from which the backbone of the functional networks was constructed using the Minimum Spanning Tree (MST) approach. Reference MSTs were computed based on the average functional connectivity matrix for either ALS‐pure motor or bvFTD, against which the networks of ALSci/bi were compared. Post‐hoc, measures of global and local network topology were compared (i.e. tree hierarchy, leaf fraction, diameter and betweenness centrality).

**Result:**

At baseline, ALSci/bi patients’ theta‐band brain networks resembled those of ALS‐pure motor more than those of bvFTD. At follow‐up, ALSci/bi patients appear to shift towards the bvFTD group in the beta band. This was reflected by the fact that ALSci/bi patients moved away from ALS‐pure motor patients overtime and their beta‐band networks resembled those of bvFTD at follow up. No significant differences in global or local network topology were found.

**Conclusion:**

Our findings suggest that brain networks of ALSci/bi patients move along the ALS‐bvFTD spectrum over time and become closer to bvFTD‐like topology.

